# Enhancing quality healthcare in Nigeria through medical laboratory services: A review

**DOI:** 10.1097/MD.0000000000036869

**Published:** 2024-01-12

**Authors:** Abdulrahman Abdulbasit Opeyemi, Emmanuel Ifeanyi Obeagu, Abdulwasiu Oladele Hassan

**Affiliations:** a Department of Medical Laboratory Science, Achievers University Owo; b Department of Medical Laboratory Science, Kampala International University, Uganda.

**Keywords:** health care, laboratory, medical laboratory scientist, medical laboratory services, Nigeria

## Abstract

This article explores the pivotal role of medical laboratory services in enhancing the quality of healthcare in Nigeria. Medical laboratory science is a comprehensive field that involves a diverse array of diagnostic and analytical procedures. These procedures are of utmost importance in the provision of patient care, the early diagnosis of diseases, and the promotion of public health. The article elucidates the progression of medical laboratory services in Nigeria, tracing the transformation from the role of laboratory assistants to that of medical laboratory scientists. It underscores the significance of these services in informing healthcare decision-making. The essay also discusses the diverse obstacles encountered by the medical laboratory profession in Nigeria. The issues encompass insufficiencies in infrastructure, obsolescence of equipment, absence of a coherent policy framework, slow workforce expansion, persistent labor strikes, and a scarcity of trained specialists. The aforementioned issues not only impede the effectiveness of laboratory services, but also have extensive ramifications for healthcare provision throughout the nation. In order to address these difficulties and improve the standard of healthcare, the essay presents practical solutions and a thorough strategy. Furthermore, it underscores the significance of augmenting financial resources, mitigating corruption, and tackling wage inequalities in order to effectively retain medical laboratory specialists. The action plan is structured into distinct phases, each delineated by specified dates and delineating the duties of various stakeholders, such as government entities, healthcare establishments, professional associations, and diagnostic enterprises.

## 1. Introduction

Medical laboratory science is the study and application of human or animal tissue analysis, bodily fluid analysis, excretion analysis, biological production, and equipment design and fabrication for medical laboratory diagnosis, treatment, and research. In addition to Clinical Chemistry (Chemical Pathology), Haematology and Blood Transfusion Science, Forensic Science, Histopathology, Molecular Biology, Laboratory Management, and any other related subject that may be approved by the appropriate authorities, it encompasses Medical Microbiology (bacteriology, parasitology, virology, and mycology).^[1]^

Beginning with the medical laboratory assistant, progressing to the medical laboratory technician (Lab Man), medical laboratory technologist, and finally the medical laboratory scientist, Nigeria medical laboratory services have grown. More than thirty Nigerian institutions now provide medical lab technician training, having partnered with various health schools to bring the programme from the UK. The Nigerian Institute of Medical Laboratory Technology (IMLT) was superseded as the regulatory body by the Medical Laboratory Science Council of Nigeria (MLSCN) by the Act of 2003. The 3 professional cadres recognized by the MLSCN (2003) in the Act are the Medical Laboratory Assistant, Medical Laboratory Technician, and now Medical Laboratory Scientist. Yet, depending on the organization, there are more staff members that work in a medical laboratory, such as pathologists, officers in charge of laboratory management of information, secretaries, ICT offices, and phlebotomists.^[2]^

Scientific data supporting patient care is provided to physicians, nurses, and other healthcare professionals by medical laboratory testing, making it an essential part of high-quality healthcare. This specialty helps identify risk factors for disease development, early disease detection, planning strategies for disease management, safe and effective treatment option selection, monitoring of treatment response, identification of threats to patient safety and public health, protection of the blood supply and recipients of transplants from harmful pathogens, adverse reactions to blood transfusions, and drug abuse testing in order to enhance clinical care and ensure public safety.^[2]^ Medical laboratories provide information and services that maximize the efficient delivery of care in today advanced health care system by making sure that the proper investigation in the laboratory is carried out on the right individual at a suitable moment and generating accurate reports of investigation that guide suppliers of samples, typically healthcare workers, to form the proper diagnostic and therapeutic choices while utilizing the proper level of health care. Medical professionals can make reasonable, empirically supported decisions about a patient diagnosis or course of therapy with the help of laboratory tests. Medical laboratory services choose the best, least invasive, and most cost-effective method to deliver the test results that are used in clinical decision-making. Additionally, it immediately affects a number of elements of patient care, such as the duration of the hospitalization, the safety of the patient, the use of resources, and the pleasure of the client.^[3]^

Having realized the emergence of medical laboratory science, it is important to emphasize that medical testing being conducted by the professionals in medical laboratories across the Nigerian health sector is a critical component of healthcare because it provides essential data for effective patient care, pathogen detection, disease surveillance, and response.^[4]^ As such, it is important to review how such services can be enhanced to improve the quality of health care services in the country. The rationale for this review is the essential function of medical laboratory services within the healthcare sector, with a specific focus on Nigeria. These services play a crucial role in the identification, surveillance, and treatment of many health conditions. The article elucidates the various obstacles encountered by professionals in this field and the broader healthcare system. By effectively tackling these aforementioned difficulties, the primary objective is to enhance the caliber of healthcare services in Nigeria, thereby guaranteeing superior patient care and overall health results. The text also discusses several important inquiries: What are the obstacles encountered by medical laboratory services in Nigeria? What impact do these problems have on the overall quality of healthcare in the nation? What potential local strategies can be employed to augment the medical laboratory profession? What are the potential measures and approaches that might be used to enhance the quality of healthcare by means of medical laboratory services? This study also seeks to illuminate the challenges and opportunities inherent in the medical laboratory profession, as well as its influence on healthcare quality in Nigeria. The objective of this initiative is to present potential resolutions and implementable strategies that encompass a range of stakeholders, such as governmental entities, healthcare institutions, practitioners, and individuals seeking medical care. The primary objective is to improve the caliber of healthcare services in Nigeria through the reinforcement of medical laboratory services and the resolution of prevailing obstacles.

## 2. Role of medical laboratory services in improving quality health care

Since Nigeria attained independence in 1960, there have been several times when improvements to healthcare services have been made.^[5]^ Nigeria succeeding governments have created a number of National Development Plans (NDPs) to address the country development challenges at different points in time.^[6]^ One of the keys turning points in the NDPs for the health sector was the growth of healthcare facilities inside towns and villages made possible by the Basic Health Service Scheme. Three tiers of public-sector care were established for the delivery of healthcare services by the NDPs. Primary, secondary, and tertiary healthcare are these 3 tiers. This structure thus reflects the 3 tiers of Nigerian government: local, state, and federal.^[7]^ Even though there were significant improvements during this time, there were still obvious shortcomings such a lack of a clear policy framework, poor resource generation, and slow labor force expansion. The industry is now dealing with issues such as ongoing strikes by medical personnel, antiquated hospital infrastructure, subpar lab equipment, and a lack of finance.^[8]^ With support from private healthcare providers and the state ministries of health in each of those states, local government areas are in charge of managing the primary healthcare system. The lower tiers of primary healthcare are the village, district, and local government area. Primary care focuses on providing patients with general medical care as well as education. The secondary healthcare system is managed by the state minister of health. Primary care usually refers patients to this level of care. Numerous governmental departments provide this, the entry-level of specialized services. In addition to many other things, Omoruan et al^[9]^ list laboratory and diagnostic services as well as rehabilitation as part of the state primary health care system. Primary healthcare that is tertiary is offered by specialty and teaching hospitals. At this level, the federal government works with nonprofit organizations as well as private practitioners.^[10]^ Secondary and tertiary care is for treating more serious illnesses that require specialized knowledge and closer monitoring of the patient health. Despite all of these obstacles, the medical laboratory profession in Nigeria remains vital to the country healthcare system.

Medical laboratory science is an integral part of the Nigerian health care system and forms the basis of diagnostic medicine.^[11]^ Healthcare includes the diagnosis, medication, and prevention of illnesses, injuries, and other impairments of both mind and body in humans. It is provided by medical professionals who work in allied health, medical laboratory science, nursing, pharmacy, medicine, and other related professions. Public health, primary care, secondary care, and tertiary care are all included in its scope.^[1]^

The duties of a medical laboratory scientist are varied, including the prompt delivery of reliable laboratory results, the supply of adequate patient information, and the observation of therapy responses.^[12]^ Keeping track of the emergence and spread of infectious and hazardous microorganisms; assisting in the selection of efficient preventative measures for big, widespread diseases; deciding on the appropriate medical laboratory tests to be performed at each time; ensuring proper calibration methods of the various specimens required; performing equipment; standardizing and validating laboratory practices; deciding on the priorities for medical diagnostics and allocating resources; ensuring critical diseases surveillance; crucially influencing clinical and societal health decisions; validating prognosis; helping to the health systems’ quality assurance and conducting potential laboratory studies for advancement and cost-cutting are all vital roles played by the medical laboratory scientists.^[13]^

A system of coordinated healthcare interventions and patient care plans used throughout the course of a disease life cycle is known as disease management (DM). The laboratory provides DM support in a variety of ways, including Disease detection, disease prediction, disease identification, treatment, and monitoring and compliance.^[12]^

As the COVID-19 pandemic in Nigeria demonstrated, medical laboratory scientists were responsible for a wide range of tasks, including detection, diagnosis, surveillance, and control of disease. Medical laboratory experts from all over the world were able to identify the disease causative agent at the beginning of the epidemic, a betacoronavirus that the World Health Organisation (WHO) subsequently designated SARS-CoV-2.^[14]^ Understanding a virus phylogenetics and properties is essential to stopping its propagation and improving medical care. An important part of medical laboratory operations in the COVID-19 response is the creation of quality control programmes to ensure the accuracy of test results, validate testing protocols, find and develop vaccines, monitor and notify about diseases, and monitor hospitalized patients with more severe COVID-19-induced complications through biochemical and serological monitoring.^[11]^ Medical laboratory scientists have advised on government containment policies in addition to supporting sterilization, disinfection, and sterilization monitoring in a facility.^[15]^ Additionally, they have put in place effective disease surveillance systems that allow them to react swiftly to outbreaks and stop their disastrous spread. Because of their combined technological and intellectual capabilities, laboratory staff play a unique role in disease surveillance and control through a variety of laboratory tests and research.^[16]^

The following could be used to sum up medical laboratory science contribution to high-quality healthcare in the new Nigeria: A few of the subject matter covered include public health preparedness and response, preventing illnesses, control, and surveillance, integrated handling of data, reference and specialized testing, environmental health and protection, policy formulation, public health-related research, education and training, collaborations, and communication.^[17]^

1. Provide analytical data in a timely and accurate manner to support communicable, infectious, genetic, and chronic disease management as well as environmental exposure prevention. This could entail testing for immune status, antibiotic resistance, inherited neonatal metabolic disorders, environmental toxins, heavy metals like blood lead, and more. It could also entail identifying outbreaks and other noteworthy public health events by identifying and characterizing the disease-causing agents and their source. By using population-based surveillance to find disorders that are crucial to the public health and to guide programming decisions, congenital illness diagnoses and treatments can be expedited for infants. tracking of high-risk and/or low-prevalence diseases such botulism, rabies, influenza, and antibiotic-resistant tuberculosis, and controlling infectious or environmental diseases requires research and study when testing in the private sector is not feasible.
2. Serve as a conduit for scientific data and information to support public health initiatives through the use of standardized data formats, policy influence, involvement in statewide disease reporting networks, data capture from laboratories essential for public health analysis and decision making, including trend and sentinel event detection, collaboration with state and local governments, and connection to the CDC and other global and national surveillance databases.
3. Act as centers of excellence for biological and chemical issues relevant to public health, utilizing their expertise, contacts, and assets to: Assist in the identification and monitoring of novel and emerging infections; Verify unusual test results from laboratories; Verify the results of tests carried out by different laboratories; Provide reference services to labs that may not be able to identify disease agents with sufficient precision that are important for public health.
4. Exercise leadership in advancing laboratory improvement in areas of public health significance by: Encouraging partner laboratories to participate in quality improvement programmes through proficiency testing, training, and consultation; creating and managing statewide laboratory improvement programmes to guarantee the accuracy of laboratory data used for environmental monitoring and communicable disease surveillance and control; encouraging safe laboratory practices through consultation, education, and training; evaluating and enhancing the State Public Health Laboratory System through the implementation of the Laboratory System Improvement Programme (L-SIP); and directing the development and enforcement of laws and regulations that advance laboratory improvement.
5. Contribute to the creation of state and federal health policies by: Developing scientific evidence to support public health policies and legislation; tracking the effects of public health laboratory practices on health outcomes; acting as resources, centers of expertise, and references for biological, chemical, and issues of public health significance; taking part in the development and review of standards pertaining to the performance and operation of laboratories conducting public health testing; and promoting the application of sound reasoning in the application of laboratory science and system infrastructure sustainment.
6. Trough developing, assessing, and implementing new technologies and methodologies; collaborating with other public health disciplines to conduct clinical and translational science innovations; collaborating with academic institutions to conduct public health systems and service research; and collaborating with other public health organizations to develop, evaluate, and implement new technologies and methodologies are some of the ways in which medical laboratory scientist conduct research to strengthen and broaden the scientific and policy.
7. Make sure that everyone has access to training and education by sponsoring opportunities for management and leadership development, taking part in the training of scientists from both home and abroad, working with academic institutions to provide experiential learning opportunities, and providing funding for ongoing education in the area of laboratory practice.
8. Utilise information technology for robust connectivity; emphasize the importance of laboratories’ contributions to public health; maintain a strong communication plan connecting all system partners; involve both traditional and nontraditional partners; and coordinate activities with the assistance of a laboratory programme advisor (i.e., laboratory system coordinator) to support their respective state public health laboratory systems.

These are some of the areas in which laboratory services in Nigeria are guiding healthcare decisions and interventions to improve public health safety, aside from the routine clinical diagnosis that is conducted across hospital laboratories and individual laboratories across the nation to guide clinical outcome, treatment, and care.

## 3. Challenges facing medical laboratory practice in Nigeria

Laboratory services whose results are the basis of 60%–70% of important clinical decisions^[18]^ suffer a lot of setbacks in the context of Nigeria health care delivery system. The challenges of inadequate infrastructure, outdated equipment, persistent lack of a clear policy framework, the slow expansion of the labor force, ongoing strikes by medical personnel, and the shortage of qualified laboratory professionals, and workforce shortages are not mere obstacles; they are crippling issues that need immediate attention required to enhance capacity of the laboratory services in delivery quality health care to the citizen.

### 3.1. Inadequate infrastructure

The presence of deteriorating healthcare systems has contributed to the emergence of medical tourism. The large number of people—about 5000 per month—who choose to receive medical care elsewhere serve as an example of this phenomenon. Consequently, the Nigerian economy experiences an annual loss of approximately 1.2 billion US dollars due to the outflow of funds towards medical tourism.^[19]^ Nigeria placed 96th out of 195 countries in the evaluation by the global health security index with a score of 37.8. The global average score provided in this assessment was 40.2 out of 100. The laboratory system capacity received a score of 50, which was lower than the average score of 54.4.^[20]^ This can be attributed to insufficient infrastructure that could potentially improve the capabilities of diagnosis and treatment. During the COVID-19 pandemic, medical laboratory scientists have played a crucial role in diagnosing individuals and conducting disease surveillance, despite facing significant risks to their own lives. However, they have encountered challenges such as inadequate infrastructure, shortages of essential facilities, difficulties in importing large diagnostic kits, delays in setting up testing facilities, misdiagnosis of COVID-19 due to insufficient training or reliance on unqualified personnel, unstable power supply, and a lack of political support for medical laboratory practices. The lack of access to locally produce testing kits and the lack of inclusion of medical laboratory components in the COVID-19 vaccination process has been observed.^[21]^

### 3.2. Outdated equipment

The presence of insufficient and outdated equipment in Nigerian hospitals and laboratories has been a significant factor in the deteriorating condition of these facilities. This issue is particularly pronounced in rural communities, where functional laboratory capabilities are lacking, leading to inefficient diagnostic processes. Even in areas where such capabilities exist, challenges such as inconsistent supply of laboratory reagents, inadequate power supply for proper reagent storage, and limited resources for training scientists on the operation of newly acquired laboratory equipment persist. These obstacles collectively impede the efficient functioning of laboratories.^[22]^

### 3.3. Persistent lack of a clear policy framework

Health governance refers to the administrative framework of the health system that is primarily focused on the policymaking and government-led steering functions. Its main objective is to achieve the national health policy goals by ensuring the effective delivery of health services and ensuring the achievement of universal health coverage.^[23]^ The healthcare system in Nigeria is primarily governed by the federal government and the ministries of health. However, this centralized approach has been found to be susceptible to issues such as corruption, political favoritism, and bureaucratic procedures, which can hinder the timely implementation of activities within the health sector.^[24]^ The aforementioned approach has consistently resulted in the annual allocation of the national health budget in Nigeria falling below the 15% benchmark set by the 2001 Abuja declaration, which stipulated that 15% of the national budget should be allocated to health. As of now, the allocation barely surpasses 7% of the nation overall budget.^[25]^ However, the implementation of this strategy has inadvertently contributed to several forms of corruption that hinder the effective functioning of the Nigerian health system. These forms of corruption include improper financial ties, fraudulent invoicing and claims, embezzlement and diversion, absenteeism, informal settlements, and the proliferation of counterfeit medical products.^[26]^

### 3.4. The slow expansion of labor force

The availability of competent health workers in Nigeria is inadequate, mostly due to both shortages and an inequitable distribution of appropriate health workforce cadres that disproportionately favors metropolitan regions. Policy adoption and implementation difficulties were influenced by a limited capacity for policy discourse and intricate coordination systems.^[27]^ The presence of unequal distribution of training institutions and trained health workers, together with the occurrence of high attrition resulting from inadequate management and development of Human Resources for Health, are substantial contributing factors. The lack of strong connections between human resources requirements and production has led to significant deficiencies in the essential health workforce, namely in the realm of primary health care.^[28]^ Despite the gradual growth of the workforce in the healthcare industry, it remains imperative to address the personal concerns of laboratory professionals. These concerns have led to a number of legal disputes and allegations. For instance, the Abuja branch of medical laboratory scientists in the country has claimed that there is a deliberate effort to gradually eliminate their members from tertiary hospitals. They argue that chief medical directors of these hospitals have been intentionally refusing to hire medical laboratory professionals in accordance with the needs analysis. This is in contrast to other professional groups in hospitals who are consistently being employed and replaced.^[29]^

### 3.5. Ongoing strikes

Frequent occurrences of industrial action within the healthcare sector, including healthcare worker strikes, are a common phenomenon in Nigeria. These strikes have significantly impacted several facets of healthcare service delivery within the country.^[30]^ The healthcare system in Nigeria has witnessed a strike by Medical Laboratory Scientists who are affiliated with the Joint Health Sector Unions. This industrial action has been primarily driven by concerns around compensation levels and emoluments, leadership positions within teaching hospitals, and the selection of the Minister of Health.^[31]^ It is imperative to emphasize that patients have significant hardships during industrial actions, as there is a notable decrease in operational activity within the healthcare system in Nigeria during such periods. According to the findings of a study conducted by,^[32]^ a cumulative number of 42 instances of strike activities were reported, with an average occurrence rate of approximately 2 strikes per year. Notably, the Joint Health Sector Union was responsible for more than half (58.1%) of the entire duration of these strikes.

### 3.6. The shortage of qualified laboratory professionals, and workforce shortages

Nigeria had 40,000 medical laboratory scientists, according to research from.^[33]^ Considering that there are 200 million Nigerians in total, this suggests that there is 1 medical laboratory scientist for every 5000 people in Nigeria. Despite the existing deficit, the emigration of Medical Laboratory Scientists from Nigeria to Europe, the Middle East, and North America exacerbates the issue.^[34]^ According to a study by,^[35]^ the nation experienced a loss of around 906 medical laboratory scientists due to human capital flight in 2022 alone. The scarcity of scientists in Nigeria may have adverse effects on laboratory services, including increased workloads for professionals, delays in obtaining test results, resulting in delayed treatment and diagnosis, and a potential gap in the provision of high-quality healthcare services. Furthermore, it is evident that Nigeria is in dire need of a substantial increase in the number of healthcare professionals in order to attain the targeted level of population coverage.

## 4. Local promising solutions to enhancing quality healthcare in Nigeria through medical laboratory services

The success of modern healthcare delivery in Nigeria and around the world depends on the accuracy and efficacy of diagnostic services offered by professionals in medical laboratories. Medical laboratory services’ practical skills and knowledge are essential to the provision of patient care and the improvement of their quality of life. They carry out a range of scientific and laboratory tests that are crucial to the diagnosis and treatment of illnesses; thus, it is vital that their actions be significantly improved.^[34]^

**Figure 1.**
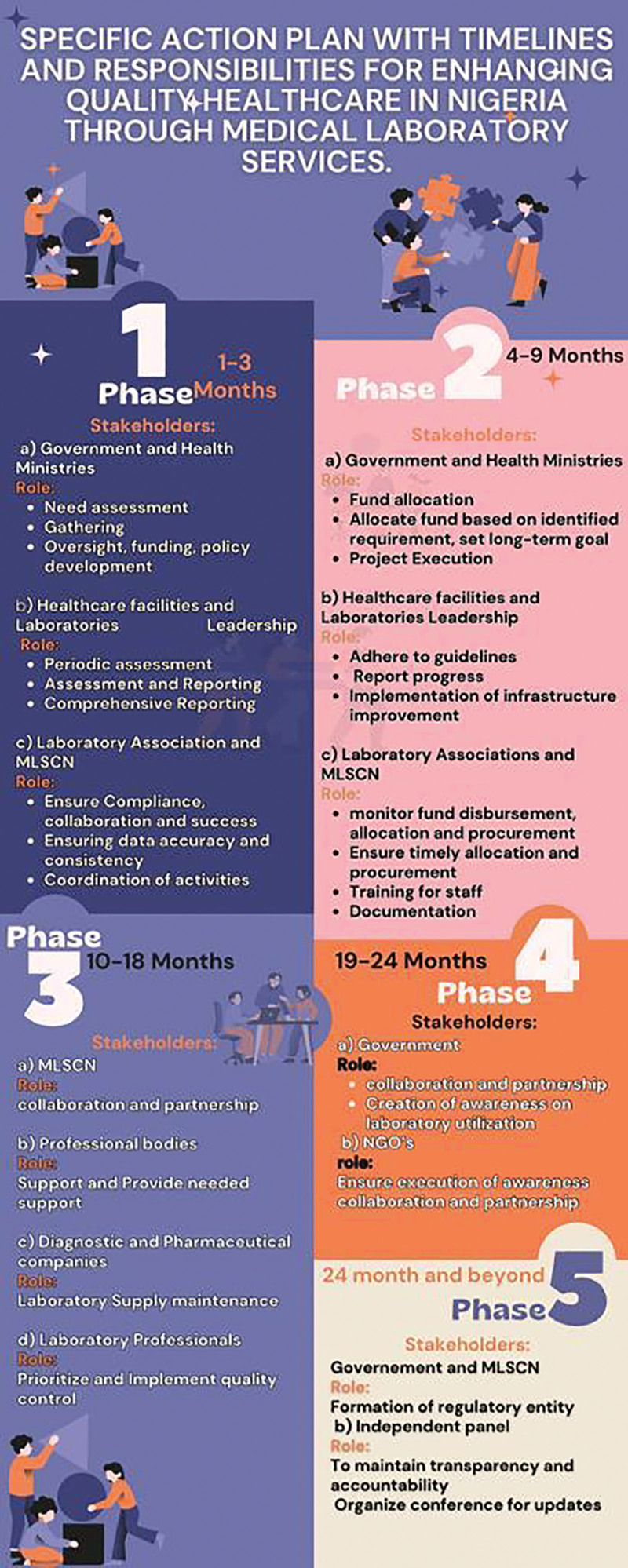
Action plan to enhance laboratory services.

Figure 1 shows Action plan to enhance laboratory services and Table 1 shows Some indices of laboratory testing conducted in Nigeria. The Medical Laboratory Science Council of Nigeria should mandate that all medical laboratory scientists employed in hospitals, laboratories, and education-based sectors enroll all of their quality assurance officers in continuing education and refresher courses on productivity and quality management systems in order to enhance professionalism and lower the costs associated with providing effective healthcare in Nigeria.^[36]^ Tax reduction can be implemented by the government on staff that have certain requirements for refresher courses to encourage others from enrolling; adequate consideration must be given to scientists in rural communities by the provision of free web-based design learning, as seen on the MLSCN site. However, courses must be tailored to current trends in diagnosis to meet international best practices. There can also be collaboration between accredited university communities in the form of an exchange programme that would facilitate idea sharing, training, and retraining among scientists in such institutions, while the management supports logistics and transportation either by deducting the 1% salary of individuals who wish to enroll for such a purpose for a period of 4 months prior to the event.

**Table 1:** Some indices of laboratory testing conducted in Nigeria.

Year	Cases	Testing conducted through laboratory service in Nigeria	Reference
	Viral load monitoring	350,625	^[42]^
Between July 2021 to June 2022	HIV testing	**>**7000,000	^[43]^
Per annum	TB	≈ 591, 277	^[44]^
-	Malaria	68,000,00	^[45]^
-	HBV and HCV	20,000,000	^[46]^
2019	Yellow fever	3547	^[44]^
2017	Cholera	1558	^[47]^
2023	Meningitis	1686	^[48]^
Till date	COVID-19	266,675 confirmed	^[18]^

Regarding the provision of financial resources, an increase in the budgetary allocation to the health sector will facilitate the accomplishment of numerous objectives. The necessity for the Nigerian government to augment its healthcare budget, as previously mentioned, is of utmost significance. However, it is important to duly consider various additional elements in order to ensure the appropriate implementation and allocation of funds for the intended purpose. The initial step entails ensuring compliance by the Nigerian government, ministries of health, and hospital management with the court directive that grants medical laboratory scientists the authority to assume complete leadership of laboratory services at different levels of the healthcare sector. This is imperative due to the favorable court rulings in support of the laboratory professionals.^[31]^ It is hypothesized that granting individuals such autonomy would enable them to establish sufficient infrastructure that would effectively enhance their work by allocating suitable financing to the laboratory. Nevertheless, it is imperative to introduce a range of anti-corruption initiatives within hospitals and laboratories nationwide. These measures should incorporate anti-corruption agencies as an integral component of the healthcare system. Additionally, the deployment of technologies such as electronic medical records or library information systems (LIS) should be employed to minimize the use of cash transactions within the health sector. Anti-corruption media campaigns should also be implemented, alongside the public disclosure of resource allocation and expenditure in healthcare. Furthermore, the establishment of a whistleblower policy to incentivize individuals who expose corrupt practices, as well as a reward system to encourage positive behavior within the health sector, should be considered.^[26]^

In relation to the phenomena of strike action and brain drain, it is noteworthy to mention that as of the year 2015, the median annual wage for Medical Laboratory Scientists and Medical Laboratory Technicians in the United States varied based on geographic area, amounting to approximately $41,420 and $60,520, respectively. When converting the amount of $60,520 to the current exchange rate of around $806.77 per dollar, the resulting value is approximately $48,825,720 per year, equivalent to approximately $4.068 million every month. In contrast, as of 2023, the median monthly income for medical laboratory scientists in Nigeria is approximately $150,000,000, in spite of the prevailing inflation rate in the country.^[33]^ In addition to the palliative wages offered by the government, it is imperative to contemplate salary increments for healthcare workers. This would serve as a significant motivating factor for them to stay in the country, especially considering the minimal disparity in salaries between the Nigerian healthcare sector and other regions to which they are migrating.

To enhance the laboratory workforce in primary health care facilities and promote equitable access to high-quality diagnostic services, it is imperative for the Medical Laboratory Science Council of Nigeria (MLSCN) to establish effective partnerships with the federal government. This collaboration should prioritize the mandatory placement of interns and National Youth Service Corps members in these areas while ensuring that they receive equitable compensation comparable to their counterparts in urban regions. Furthermore, it is imperative to ensure that appropriate welfare measures, such as providing suitable accommodation, are readily accessible to individuals engaged in call-duty services. Other professionals could be deployed to their respective local environments in order to reduce lodging expenses and enhance diagnostic capabilities. The fundamental objective should involve a reorganization of both the internship programme and the National Youth Service Corps to effectively enhance the diagnostic workforce inside the nation. Furthermore, it is imperative for the government to demonstrate a willingness to promptly incorporate these individuals into the civil service programme before exploring alternative measures to augment the national workforce.

The implementation of a bottom-up strategy is imperative within the healthcare sector of Nigeria, as it is essential for the development of comprehensive policies that address the needs of individuals residing in rural communities. By adopting this method, all stakeholders would be provided with an equitable platform, rather than burdening the bulk of responsibilities on individuals located in Abuja. Therefore, it is imperative to implement the second national strategic health development plan for the period of 2018 to 2022, which takes into account the aforementioned methodology. This implementation should involve the active participation of key stakeholders in the health sector as well as various levels of government.^[37]^

It is imperative to support domestic diagnostic manufacturing companies within the state in order to enhance the accessibility of laboratory equipment nationwide. Concurrently, the Medical Laboratory Science Council of Nigeria (MLSCN) plays a crucial role in certifying and standardizing the goods of these companies. It is imperative to ensure the provision of adequate funds for research endeavors aimed at enhancing diagnostic capacity. Both individuals and organizations that are prepared to contribute to this cause should have access to research grants. The allocation of these grants should be determined based on the criteria of merit, purpose, worthiness, and the potential impact on diagnostic capacities. Furthermore, it is vital to give precedence to public-private partnerships by considering their importance and heeding the advice of laboratory specialists, who are the primary recipients of the outcomes derived from these joint efforts. The promotion of public-private partnerships (PPPs) is crucial in reducing the government burden in terms of outreach provision and addressing funding constraints.^[38]^

## 5. Action plan with timelines and responsibilities for enhancing quality healthcare in Nigeria through medical laboratory services

It is important to recognize the workforce of laboratory professionals and the dire need to implement actionable plans that would enhance their effective performance in delivering quality laboratory services that would improve healthcare delivery in the Nigerian healthcare context. In achieving such plans, critical stakeholders like the government and health ministries in the country, healthcare facilities and laboratories, MLSCN and professional bodies, diagnostic and pharmaceutical industries, non-governmental organizations, patients and communities, and must team up as one to ensure the sustainability and implementation of the following actionable plans:

### 5.1. Phase 1: Assessment and planning (1–3 months)

Healthcare planning and assessment are widely recognized as essential elements of health system governance due to their role in enabling decision-makers to shape and guide the provision of health services.^[39]^ This function is expected to gain greater significance as health systems in Nigeria confront progressively intricate challenges that necessitate innovative approaches. This is due to the fact that during this particular phase, stakeholders will have the opportunity to identify unfulfilled requirements within the laboratory services of the Nigerian healthcare system. Consequently, they can establish a suitable and efficient framework to address these needs.^[36]^ Failure to address these demands may result in the implementation of a hierarchical methodology for delivering healthcare, wherein a limited number of individuals dictate the perceived requirements of the populace, rather than accurately identifying and meeting their genuine needs.^[36]^ These particular phases must directly involve the government and health ministries, who will be charged with the responsibility of assessing the current laboratory infrastructure and needs of the laboratory through standardized grading rubrics, surveys on laboratory needs amongst various local and national healthcare facilities, or conducting focus group interviews with end users of laboratory equipment at all strata of the healthcare system in the country. All healthcare facilities and laboratories through their leadership must conduct periodic assessments within this phase on a yearly basis to identify immediate infrastructure and equipment needs and prepare comprehensive reporting for the subsequent phase, while laboratory associations and MLS must ensure compliance, collaboration, and the success of these phases at their respective facilities on a regular basis.

### 5.2. Phase 2: Infrastructure improvement (4–9 months)

During this stage, the stakeholders previously mentioned have successfully identified the necessary requirements. It is now crucial for them to ensure the availability of these requirements in order to enhance health infrastructure and enable laboratory diagnosis, thereby facilitating social and economic activities.^[40]^ In order to accomplish this objective, it is imperative for health ministries and governmental bodies to ensure the appropriate allocation of funds towards the enhancement of laboratory infrastructure which can be targeted on selective laboratories based on random selection with a long-term goal of revamping the laboratory setting within the next 5–7years, given its crucial role within the health sector. Additionally, these entities must oversee the execution of infrastructure projects, while healthcare facilities and laboratories adhere to established guidelines for implementing infrastructure improvements. Furthermore, it is essential to provide training to staff members on the utilization of new equipment and for laboratory associations and professional organizations to monitor the expeditious disbursement of funds as well as the allocation and procurement of necessary items within the designated timeframe. In order to maintain openness and accountability throughout the action plan phase, it is imperative to prioritize the documenting and recording of activities at each stage.

### 5.3. Phase 3: Quality control and training (10–18 months)

During this time, it is expected that relevant items and infrastructure required for laboratory advancement will be secured within each of the laboratory facilities in the country. The MLSCN, professional body, and diagnostic or pharmaceutical companies supplying reagents and equipment must team up in a formidable partnership to ensure the training of laboratory professionals. Considering the economic situation of the country, MLSCN can subsidize the training fees of the identified targeted facilities for an actionable plan by ensuring their integration with the requirement for license renewal in order not to charge professionals exorbitantly. Diagnostic or pharmaceutical companies must also be responsible for the maintenance of equipment and reagents, while laboratory professionals prioritize internal and external quality control systems based on established protocols in the facilities at intervals.

### 5.4. Phase 4: Access and awareness (month 19–24):

At this stage, it is imperative for governmental and non-governmental entities to collaborate in order to initiate public awareness initiatives regarding the significance of laboratory services. These campaigns aim to promote the utilization of laboratory services by patients and communities, emphasizing the importance of conducting health checks prior to indiscriminate medication intake. It is worth noting that laboratory tests can greatly contribute to the improved diagnosis and management of 6 of the ten most prevalent diseases and conditions observed in outpatient settings within hospitals.^[41]^

### 5.5. Phase 5: Sustainability and ongoing improvement beyond 24 months

It is crucial that all stakeholders who have been identified possess a designated representative who will contribute to the formation of a well-established regulatory entity. This entity will be responsible for the ongoing supervision and enhancement of the actionable plan. It is critical to include an independent panel within the sustainability team to effectively manage funding and execute actionable plans within a 24-month timeframe. Additionally, organizing conferences that provide updates on the plan cycle and encourage interactive sessions with relevant stakeholders will further enhance the implementation of sustainable plans.

## 6. Conclusion

The review has provided an in-depth analysis of the role of medical laboratory services in enhancing the quality of healthcare in Nigeria. As a critical component of the healthcare system, medical laboratory professionals play a pivotal role in diagnosis, monitoring, and disease control, making their services indispensable to patient care. However, this profession faces significant challenges that need to be urgently addressed to maintain and enhance the quality of healthcare in Nigeria.

Inadequate infrastructure and outdated equipment in healthcare facilities have hampered the efficiency and effectiveness of laboratory services. While urban areas may have relatively better-equipped facilities, rural communities often suffer from a lack of basic laboratory capabilities. This imbalance in infrastructure needs to be rectified to ensure that all citizens have access to high-quality healthcare, regardless of their location.

One of the systemic issues that needs attention is the slow expansion of the labor force in the medical laboratory profession. The shortage of qualified professionals, coupled with wage disparities, has led to a brain drain as many professionals seek better opportunities abroad. To mitigate this, wage structures for healthcare workers should be revised to make the profession more attractive and competitive both within the country and internationally.

The frequent strikes within the healthcare sector, often driven by compensation issues and leadership disputes, have disrupted healthcare delivery, causing suffering for patients. These strikes underscore the urgent need for resolution mechanisms and improved coordination in the healthcare system to ensure that such disputes do not lead to a breakdown in services.

## Author contributions

**Conceptualization:** Abdulrahman Abdulbasit Opeyemi.

**Methodology:** Abdulrahman Abdulbasit Opeyemi, Emmanuel Ifeanyi Obeagu, Abdulwasiu Oladele Hassan.

**Supervision:** Emmanuel Ifeanyi Obeagu, Abdulwasiu Oladele Hassan.

**Validation:** Emmanuel Ifeanyi Obeagu, Abdulwasiu Oladele Hassan.

**Visualization:** Emmanuel Ifeanyi Obeagu, Abdulwasiu Oladele Hassan.

**Writing – original draft:** Abdulrahman Abdulbasit Opeyemi, Emmanuel Ifeanyi Obeagu, Abdulwasiu Oladele Hassan.

**Writing – review & editing:** Abdulrahman Abdulbasit Opeyemi, Emmanuel Ifeanyi Obeagu, Abdulwasiu Oladele Hassan.
